# From medicine to fields: what the 2026 nobel prize means for agricultural biotechnology

**DOI:** 10.1007/s44307-026-00142-7

**Published:** 2026-10-19

**Authors:** Zhan-Yan Zhang, Qing-Qing Liu, B. Marambe, Barana C. Jayawardana, Ge-Fei Hao, Wen-Chao Yang

**Affiliations:** 1 Key Laboratory of Green Pesticide, School of Pharmaceutical Sciences, Center for Research and Development of Fine Chemicals, Guizhou University, Guiyang, 550025 People’s Republic of China; 2 Department of Crop Science, Faculty of Agriculture, University of Peradeniya, Peradeniya, 20400 Sri Lanka

Karl Deisseroth of Stanford University in the United States, Peter Hegemann of Humboldt University of Berlin in Germany, and Georg Nagel of the University of Würzburg in Germany jointly received the 2026 Nobel Prize in Physiology or Medicine for their discoveries related to light-gated ion channels and optogenetics. Peter Hegemann and Georg Nagel first identified channelrhodopsins in unicellular green algae and characterized them as blue-light-activated, light-gated ion channels (Nagel et al. 2002, 2003). Building on these discoveries, Karl Deisseroth successfully applied channelrhodopsins to animal neurons (Aravanis et al. 2007), advancing the development of optogenetics in neuroscience and enabling the rapid and controlled modulation of specific cellular activities using light. This light-based technology, which has become well established in animal and biomedical research, also offers new perspectives for agricultural research.

In recent years, channelrhodopsins have made a series of advances in plant signal transduction, pest control, and crop breeding research. Ding et al. found that the channelrhodopsin XXM 2.0 mediates Ca^2+^ influx into plant leaf cells upon green-light illumination, activating reactive oxygen species (ROS) bursts, defense hormone pathways involving jasmonic acid, salicylic acid, and ethylene, as well as programmed cell death (Ding et al. 2024). These responses mimic immune responses triggered by pathogen infection. In contrast, ACR1 2.0 triggers drought stress responses through anion efflux, including stomatal closure and abscisic acid accumulation (Ding et al. 2024). Brockmann co-expressed channelrhodopsin 2 (ChR2) with the insect voltage-sensitive sodium channel (VSSC) and used light pulses to precisely control channel opening, thereby establishing a light-activated high-throughput screening platform for identifying novel compounds targeting VSSC (Brockmann 2023). In addition, photopharmacological strategies incorporate photoswitches, such as azobenzene, into insecticide molecules, enabling optical control of multiple insect ion channel targets, including γ-aminobutyric acid receptors, ryanodine receptors, nicotinic acetylcholine receptors, and glutamate-gated chloride channels (Fu et al. 2023). Zhou et al. demonstrated that the light-gated anion channel ACR1 2.0 mediates anion efflux and membrane depolarization in pollen tube cells under green-light illumination. Uniform illumination reversibly inhibits pollen tube growth, whereas localized asymmetric illumination redirects pollen tube growth away from the illuminated side, indicating that anion efflux and membrane potential gradients contribute to the directional guidance of pollen tube tip growth (Zhou et al. 2021). Collectively, these findings demonstrate that channelrhodopsins provide light-based tools for investigating plant stress signaling and screening insecticidal compounds, while also offering new perspectives for crop breeding.

**Fig. 1:**
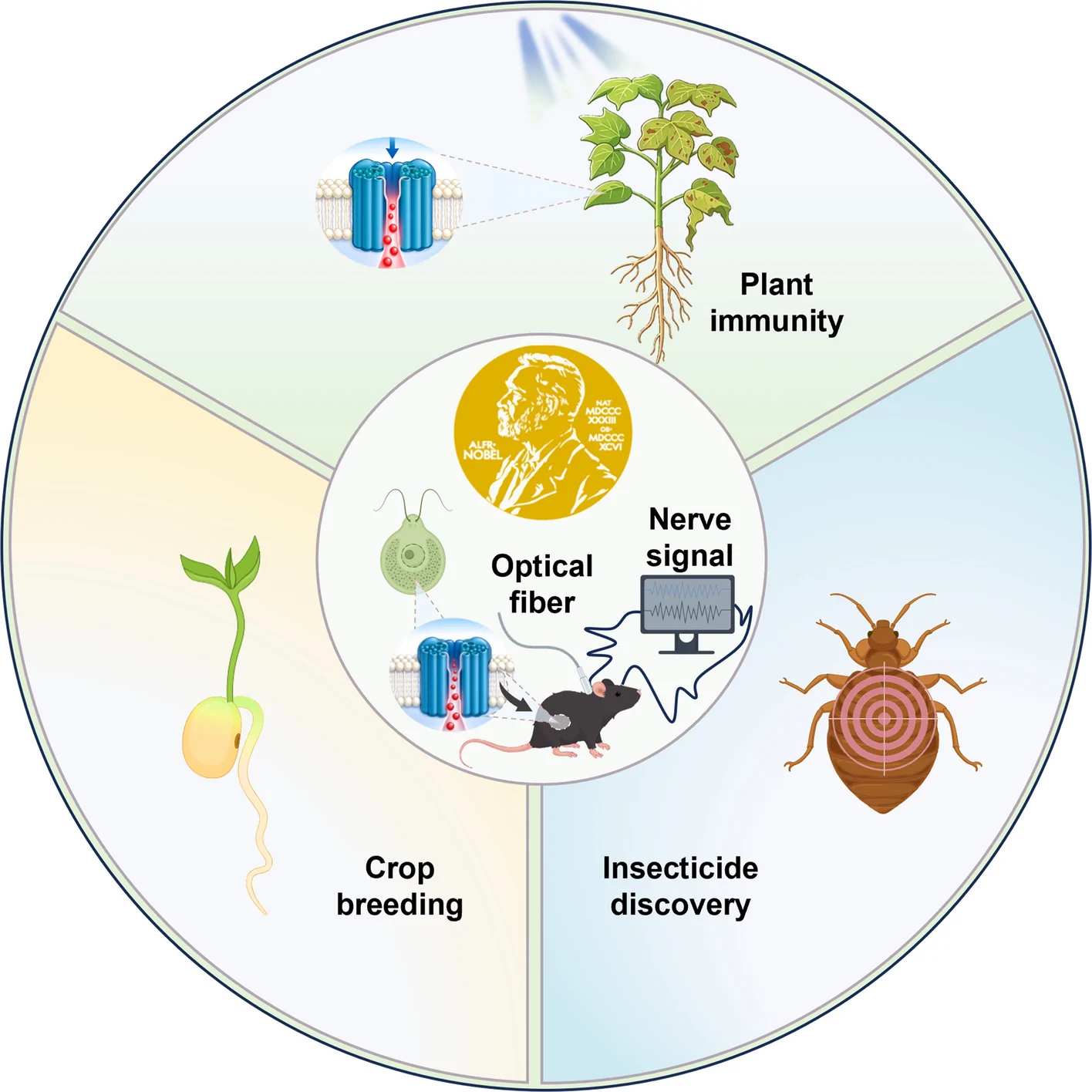
Potential agricultural applications of channelrhodopsin-mediated ion flux manipulation

In the future, channelrhodopsin-mediated manipulation of ion fluxes is expected to find broader applications in mechanistic studies of plant biology, pesticide development, and crop breeding (Fig. 1). In plant mechanistic research, XXM 2.0 and ACR1 2.0 could be used to manipulate Ca^2+^ influx and anion efflux, respectively. By varying the duration and frequency of light stimulation, researchers could compare the resulting effects on ROS production, phytohormone responses, and cellular damage. Combined with cell-type-specific expression, transcriptomic analysis, and functional validation of candidate genes, this approach could help identify key regulatory nodes linking ion signaling to defense responses. Their actual contributions to disease resistance and stress tolerance could then be evaluated through pathogen inoculation and drought stress assays. In pesticide discovery, the ChR2-assisted screening strategy for insect VSSC could serve as a model for developing functional screening systems targeting different insect ion channels. Candidate compounds could be further evaluated through electrophysiological validation and insect bioassays to confirm their activity. Integrating optogenetics-assisted screening with photopharmacological molecular design could provide complementary strategies for discovering novel insecticidal lead compounds and elucidating their mechanisms of action. In crop breeding, the observation that ACR1 2.0 regulates pollen tube growth provides a starting point for further investigating the relationships among anion fluxes, changes in membrane potential, pollen tube guidance, and fertilization. Genetic variation analysis and genome editing could then be used to validate candidate regulatory factors and determine their effects on agronomic traits, such as seed set.
